# Japanese encephalitis acquired in Australia.

**DOI:** 10.3201/eid0103.950310

**Published:** 1995


### Japanese Encephalitis Acquired in Australia

Japanese encephalitis (JE), a mosquito-borne flaviviral disease of humans and animals, is a major public health problem in Asia, where an estimated 50,000 cases occur each year. There has been concern that the range of epidemic JE may be expanding.

On April 5, 1995, an outbreak of three cases of JE was recognized in Australia. Two of the cases were fatal; all were among residents of an island in Australia's Torres Strait, which lies between mainland Queensland and Papua New Guinea. JE was confirmed in two of the patients by polymerase chain reaction (Jeffrey Hanna, Queensland Health, pers. comm.). No other cases were reported. This is the first recognized episode of JE acquired in Australia.

Control activities on the Australian island began on April 7. The community was informed about the importance of personal mosquito protection measures. In addition, larvicides were applied, and areas were fogged to kill adult mosquitoes.

The patients were all male, aged 6 to 44 years. All were hospitalized with symptoms that included fever (up to 40ºC), stiff or painful neck, headache, and abdominal pain. Two patients were unconscious at the time of admission.

Acute-phase sera showed elevated JE virus immunoglobulin M (IgM) titers. Two of the patients also had detectable levels of Kunjin and Murray Valley encephalitis virus IgM, but the JE IgM titers were significantly higher in each case.

Flaviviruses have also been isolated from the sera of each of two asymptomatic island residents. Preliminary tests suggest that these are both JE virus. Blood taken from 10 horses and 12 domestic pigs living near humans on the island was also tested. All 12 pigs and 9 of the horses had high JE titers by hemagglutination inhibition assay. Neutralizing antibody to JE virus was detectable in all the pigs and in four of the horses tested to date.

Details of the index case are as follows: The patient, a 16-year-old male, was admitted to Thursday Island Hospital on March 22, 1995. He was unconscious and was responsive only to painful stimuli. His neck was stiff, and he showed a preference for moving his right side. His illness had begun 3 days before. The day before admission he complained of abdominal pain. This patient had been mildly mentally retarded since birth and occasionally had generalized seizures but was generally healthy. He was transferred to Cairns Base Hospital, where a cerebral CT scan showed a nonenhancing hypodense lesion in his posterior right basal ganglia.

He had a leukocytosis of $17.3 \times 10^9$L, neutrophils, $15.2 \times 10^9$. His cerebrospinal fluid contained 150 leukocytes/µl with a differential count of 50% polymorphs and 50% mononuclear cells.

He had a generalized seizure and 2 days after admission, required mechanical ventilation. He never regained consciousness and died on day 17 of hospitalization (April 8).

---

Adapted from Hanna J, Ritchie S, Loewenthal M, et al. Probable Japanese encephalitis acquired in the Torres Strait. Communicable Diseases Intelligence 1995;19:206-7.

### USPHS and IDSA Collaborate on Guidelines to Prevent Opportunistic Infections in HIV-Infected Persons

U.S. Public Health Service (USPHS)/Infectious Diseases Society of America (IDSA) Guidelines for Preventing Opportunistic Infections in HIV-Infected Persons will be published in an August 1995 supplement of *Clinical Infectious Diseases*. The guidelines, which are intended for health care providers, are the result of collaboration between the Centers for Disease Control and Prevention (CDC), the National Institutes of Health, IDSA, numerous federal and nonfederal organizations, community groups, and HIV-infected persons. The guidelines are endorsed by the American Academy of Pediatrics, the Infectious Diseases Society of Obstetrics and Gynecology, and the Society of Healthcare Epidemiologists of America. Jonathan E. Kaplan, M.D. (CDC), Henry Masur, M.D. (NIH), and King Holmes, M.D., Ph.D. (University of Washington), chaired the USPHS/IDSA Prevention of Opportunistic Infections Working Group and are guest editors of the *Clinical Infectious Diseases* supplement.

CDC initiated work on the guidelines in early 1994; meetings were held in Atlanta in June and September to discuss and refine the recommendations.

The USPHS/IDSA guidelines address 17 opportunistic infections from three angles: 1) preventing exposure to opportunistic pathogens (e.g., sexual, occupational, and environmental exposure as well as exposure through pets, food, water, and international travel); 2) preventing opportunistic disease by chemoprophylaxis and vaccination; and 3) preventing disease recurrence. In this document, new recommendations were made and earlier recommendations were updated. For example, new guidelines recommend that in nonemergency situations, cytomegalovirus (CMV)-seronegative HIV-infected persons who require blood transfusions receive only

